# Risk factors for vitamin A and D deficiencies among children under-five in the state of Palestine

**DOI:** 10.1186/s13031-018-0148-y

**Published:** 2018-04-02

**Authors:** Aeysha Bushra Chaudhry, Shakoor Hajat, Najwa Rizkallah, Ala’a Abu-Rub

**Affiliations:** 1 The London School of Hygiene and Tropical Medicine, Keppel Street, London, WC1E 7HT UK; 2 UNICEF Iraq Country Office, 100 mt street, UN Compound, Erbil, Iraq; 3 Ministry of Health, Ramallah, State of Palestine

**Keywords:** Vitamin A deficiency, Vitamin D deficiency, Risk factors, Palestinian children

## Abstract

**Background:**

Vitamin A and D are essential for the proper growth and development of a child. Due to the complex political circumstances in the state of Palestine, research on micronutrient deficiency is scarce.

**Methods:**

The Palestinian Ministry of Health (MOH) and UNICEF conducted a national cross-sectional survey in 2013 after the implementation of various micronutrient supplementation and fortification programs. Risk factors for levels of vitamin A (*n* = 1054) and vitamin D (*n* = 150) were assessed among children aged 6 to 59 months using chi-square tests and logistic regression with each of the outcome variables, vitamin A and D deficiencies. A child was considered to be deficient in vitamin A and D if he/she had a serum level < 1.05 μmol/L and < 50 nmol/L respectively. Multiple logistic regression models were developed to identify independent risk factors for vitamin deficiencies.

**Results:**

The prevalence of vitamin A and D deficiency was 73.1% and 60.7% respectively. Children in Gaza were 1.34 (95%CI 0.78–2.31) and 1.96 times (95%CI 0.67–5.71) more likely to be deficient in vitamin A and D respectively compared to children in the West Bank. Anaemic children were 1.5 times more likely to be deficient in vitamin A (95%CI 1.08–2.10). Older children (> 1 year-old) were more likely to be deficient in vitamin D, and females were 2.72 times more likely to be deficient than males (95%CI 1.21–6.01). Results suggest no association between maternal education levels, feeding practices such as breastfeeding and complementary feeding and vitamin A and D deficiency. Although not reaching conventional levels of statistical significance, it was observed that children who received their vitamin drops from the MOH were more likely to have vitamin A and D deficiencies than those children receiving the supplements from the United Nations Relief and Works Agency for Palestine Refugees (UNRWA).

**Conclusions:**

Using these results, the MOH may consider specifically targeting at risk children to increase adherence to the full supplementation regimen. Further research into effective methods of service delivery by health service providers is needed including an in depth look at the UNRWA maternal counselling and supplement provision protocols.

## Background

Due to the complex political and economic situation, the population of the State of Palestine may be particularly vulnerable to micronutrient deficiencies. An estimated 25.8% of the population of 4.8 million lies under the poverty line [1]. The poverty rate varies dramatically within the country’s two main regions: the West Bank (17.8%) and the Gaza Strip (38.8%) [1]. In combination with these poor economic indicators, a World Health Organization report in 2016 demonstrated that the Palestinian Ministry of Health (MOH) faces political instability and tends to rely on donor funding for its work [2]. The population in the Gaza Strip is more likely to have poor health outcomes than the West Bank as it is currently under a blockade by land, sea and air. Furthermore, in 2014 Gaza experienced military airstrikes resulting in civilian deaths, the destruction of critical health care facilities and disruption of the food supply [2]. Accessing and providing national healthcare services, such as micronutrient supplements, may also be dependent on the presence of military checkpoints [2]. There are four primary health providers that try to implement care in these precarious political circumstances: MOH, Non-Governmental Organizations (NGOs), United Nations Relief and Works Agency for Palestine Refugees (UNRWA), and private providers. The UNRWA provides services to registered refugees with concentrated efforts in Gaza [2]. High poverty rates combined with political tensions make provision and coordination of health care by the MOH very difficult. Thus, it is critical to assess and monitor progress of state-wide programs and examine any differences in service provision between various regions.

Micronutrients such as vitamin A and vitamin D are critical for the proper growth and development of children. Due to the micronutrient’s role in bone mineralization, in its most severe form, vitamin D deficiency (VDD) can lead to rickets [3–5]. The primary source of vitamin D is UV radiation, and despite ample sunlight in the region, estimates of the prevalence of VDD among children under-five in the Middle East and North Africa range between 30% and 75% [3]. Some studies attribute this to reduced outdoor physical activity, the clothing style in the region, or to seasonal variations in a child’s month of birth (i.e. a child born in winter versus the summer) [4, 6–12].

Vitamin A, also known as retinol, is essential for cellular differentiation, which impacts growth, reproduction, the immune response and most notably visual function [13, 14]. In severe cases, vitamin A deficiency (VAD) can cause permanent blindness, and is also associated with increased morbidity and mortality from childhood infections such as diarrheal disease, respiratory infections and measles [13–15]. This may be attributable to the inverse relationship of the vitamin with levels of C-reactive protein (CRP), which tend to be elevated during infections and suppress uptake of vitamin A [14, 16]. Furthermore, VAD has also been closely linked to the presence of stunting and wasting in children [15]. Few studies have estimated the overall prevalence of VAD among children in the Middle East alone, however one study estimated the prevalence was 11% in the combined regions of the Middle East, North Africa and Central Asia [15].

Studies in the Middle East have highlighted trends in the following risk factors common in both vitamin A and D deficiencies: gender, age and maternal education. While female children have been found to be more likely to have VDD than males, [4, 17] two studies in Saudi Arabia and Iran demonstrated VAD was more common in male neonates [18, 19]. Some evidence suggests older children are more likely to have VAD and VDD as opposed to their younger counterparts [5, 13, 14]; however, there have been some studies that have observed the opposite trend [4, 20]. Additionally, evidence has suggested an inverse relationship between maternal education levels and VDD [3, 4] and VAD [16].

The role of breastfeeding and complementary feeding has also been examined [16, 21]. While exclusive breastfeeding is an adequate source of vitamin A for children under six months of age, this alone cannot be the only source of vitamin D [14, 21]. Lastly, children with VAD have been considered more likely to develop anaemia due to vitamin A playing a crucial role in red-blood cell production and iron metabolism [13, 14]. A cross-sectional study in Jordan explored a potential relationship of anaemia with VDD, yet no association was found [4].

This study examined the data collected from the Palestinian Micronutrient Survey in 2013, which assessed the supplementation program for vitamin A and D for children under-five. The main aim was to ascertain the prevalence and the risk factors for VAD and VDD among children in the state of Palestine. Moreover, the study measured the uptake of the vitamin A and D supplementation program and considered whether variations in VAD and VDD are related to the availability of supplements by different health service providers.

## Methods

### Data collection and management

The Palestinian MOH funded a national study, the Palestinian Micronutrient Survey (PMS), with technical, logistical and financial support from UNICEF, to conduct one of the first evaluations of its food fortification and supplementation programs, including the provision of vitamin A and D supplements to children from 21 days old up to 12 months of age. The survey was administered to children ages six to 59 months. Randomized cluster sampling using probability proportional to size generated 40 clusters in Gaza and the West Bank with 15 individuals selected from each cluster [22]. The researchers determined the sample size using the following formula: *n = 1.96*^*2*^ * p (1-p) *Deff ***/*** *e*^*2*^. Informed by the multiple cluster indicator survey (MICS4), the prevalence value for anaemia (*p* = 20%) was used to calculate the sample size as this was initially the main health outcome of interest in the PMS. The design effect (Deff) was 1.4 to correct any loss in precision due to choosing cluster sampling. This Deff was chosen as there is minimal variation in childhood anaemia levels in the same cluster in both the West Bank and Gaza Strip. Furthermore, the population is homogenous in regards to health conditions, culture and socioeconomic conditions within the same cluster. The formula yielded a sample size of 1200 children ages six to 59 months. The recruitment of the children took place at clinics for women and their children.

The field workers received verbal informed consent from the mothers of the children to provide information regarding the child’s health and to collect blood samples. The analysis of the blood samples was conducted by teams at Central Public Health Lab West Bank and PHD-Laboratories. The laboratory ensured the quality of its methods by repeated analyses (*n* = 6–10) of the same sample to determine the inter-assay coefficient of variance (IACV) [22]. The study found the IACV for retinol and 25 OH-cholecalciferol to be 3.31 and 3.9 respectively, where values less than 10 are considered robust [22]. In addition to blood serum tests, data were collected on demographic, socioeconomic factors and nutritional practices that enable the assessment of risk factors for these micronutrient deficiencies.

### Survey data

The risk factors that were examined in this study fall into the following categories: demographic, geographic, socio-economic, and frequency of supplement provision. There were also data on the availability of the vitamin supplements by the particular health service providers. The relationship between anaemia and vitamin A and D deficiency was also considered by assessing mean levels of haemoglobin (Hb) in children. Anaemia was classified as a child having an Hb level less than 11.0 g/dl [22].

### Analysis

While the sample size was 1200, only 1054 samples of vitamin A were used for this analysis. Quality control teams at the district level rejected 108 samples as they were clotted, broken or opened. The Central Public Health Laboratory (CPHL) received the remaining 1092 samples and rejected an additional 38 for the aforementioned reasons. Vitamin D samples were only taken form 150 children as vitamin D was not part of the original study proposal submitted to donors. As the costs of the kits for analyzing VDD was high, the micronutrient survey committee could only afford to collect 150 samples.

Vitamin A was measured in ppb and was converted to μmol/L by multiplying by the international conversion factor standard, 3.491. Vitamin D was measured in pg/ml, and was converted to nmol/L by using the international conversion factor standard of 2.496. Since vitamin D levels were recorded in only 150 of the children sampled, the outcome variables were dichotomized as follows using cut-offs defined by CPHL West Bank and PHD-Laboratories [22]: Vitamin A < 1.05 μmol/L deficient/low, ≥ 1.05 μmol/L sufficient; Vitamin D: < 50 nmol/L deficient/low, ≥ 50 nmol/L sufficient. In other works of literature these vitamins have an additional categorical cut-off: Vitamin A: < 0.7 μmol/L deficient and Vitamin D: < 25 nmol/L deficient.

Chi-squared tests were used to examine the association between categorical risk factors and the binary micronutrient outcomes. Fisher’s exact tests were used if any of the expected values were less than one. Logistic regression was performed to determine the odds ratios. Tests for trend were used to assess the outcomes with the ordered categorical risk factors of education and age group. Similarly, associations between vitamin deficiency and presence of anaemia were also assessed using chi-squared tests.

Multiple logistic regression models were used to assess independent effects of risk factors. Final models adjusted for core and modifiable variables, based on prior hypotheses rather than on statistical significance. Factors were considered modifiable if an intervention could impact on them, such as a health promotion campaign could increase levels of breastfed children. Each model allowed for the clustering of the district variable. Wald tests generated the *p*-values in the final model. The data analysis software used was STATA version 14 [23].

## Results

Of the 1200 children sampled, serum level of vitamin A was recorded in 1054 children (87.8%), and vitamin D in 150 children (12.5%). Table 1 shows the prevalence of vitamin A and D deficiency.

**Table 1:** Prevalence of Vitamin A and D Deficiency in Children 6–59 months

Vitamin	Total N	Deficient N (%, 95% CI)	Low N (%, 95% CI)	Sufficient N (%, 95% CI)
Vitamin A^a^	1054	348 (33.0, 30.2–35.6)	423 (40.1, 37.2–43.1)	283 (26.9, 24.2–29.5)
Vitamin D^b^	150	9 (6.0, 2.2–9.8)	82 (54.7, 46.7–62.6)	59 (39.3, 31.5–47.2)

^a^Vitamin A: Deficient (< 0.7 μmol/L) Low (0.7–1.0499 μmol/L) Sufficient (≥ 1.05 μmol/L)

^b^Vitamin D: Deficient (< 25 nmol/L) Low (25–49.99 nmol/L) Sufficient (≥ 50 nmol/L)

### Vitamin A results

The mean level of vitamin A was 0.900 μmol/L (StDev: 0.392) and 73.1% (95%CI 70.5–75.8%) of the children were considered deficient (< 1.05 μmol/L). Table 2 shows the unadjusted associations between risk factors and the presence of VAD.

**Table 2:** Unadjusted Associations between Risk Factors and Vitamin A Deficiency

Risk Factor	Subcategory	N (%)	Deficient N (%)	OR^a^	95% CI	*p*-value^b^
Total	*Total*	1054(100)	771(73.1)	N/A	N/A	N/A
Age Group	< 1	131(12.5)	94(71.8)	1		Test-for- trend: 0.299
	1–2	268(25.5)	192(71.6)	0.99	0.63–1.58	
	2–3	270(25.7)	199(73.7)	1.10	0.69–1.76	
	3–4	200(19.1)	141(70.5)	0.94	0.58–1.53	
	4+	180(17.2)	140(77.8)	1.38	0.82–2.31	
Gender	Male	567(53.8)	414(73.0)	1		0.916
	Female	487(46.2)	357(73.3)	1.01	0.77–1.33	
Mother’s Education	Illiterate/Elementary	59(5.7)	47(79.7)	1		Test-for- trend: 0.008
	Preparatory/Secondary	657(63.3)	493 (75.0)	0.77	0.40–1.48	
	Diploma/BA/Postgrad	322(31.0)	218(67.7)	0.54	0.27–1.05	
Province	West Bank	569(54.0)	417(73.3)	1		0.914
	Gaza Strip	485(46.0)	354(73.0)	0.99	0.75–1.29	
District	Jenin	71(6.7)	50(70.4)	1		< 0.001
	Tulkarm	45(4.3)	37(82.2)	1.94	0.78–4.87	
	Nablus	68(6.5)	47(69.1)	0.94	0.46–1.94	
	Qalqiliya	30(2.8)	18(60.0)	0.63	0.26–1.54	
	Salfit	15(1.4)	14(93.3)	5.88	0.73–47.6	
	Tubas	11(1.0)	4(36.4)	0.24	0.06–0.91	
	Ramallah &Al-Bireh	83(7.9)	60(72.3)	1.10	0.54–2.21	
	Jericho & Aghwar	15(1.4)	14(93.3)	5.88	0.73–47.6	
	Jerusalem	30(2.8)	20(66.7)	0.84	0.33–2.10	
	Bethlehem	29(2.8)	29(100.0)	N/A	N/A	
	Hebron	114(10.8)	74(64.9)	0.78	0.41–1.47	
	South Hebron	58(5.5)	50(86.2)	2.63	1.06–6.48	
	North Gaza	86(8.2)	56(65.1)	0.78	0.40–1.54	
	Gaza	173(16.4)	131(75.7)	1.31	0.71–2.43	
	Deir El Balah	68(6.5)	51(75.0)	1.26	0.60–2.66	
	Khan Yunis	96(9.1)	68(70.8)	1.02	0.52–2.00	
	Rafah	62(5.9)	48(77.4)	1.44	0.66–3.15	
Type of Community	City	460(43.6)	347(75.4)	1		0.058
	Village	313(29.7)	224(71.6)	0.82	0.59–1.13	
	Camp	263(25.0)	183(69.6)	0.74	0.53–1.04	
	Bedouin camp	18(1.7)	17(94.4)	5.54	0.73–42.1	
Has the child ever been breastfed?	Yes	1026(97.3)	753(73.4)	1		0.283
	No	28(2.7)	18(64.3)	0.65	0.30–1.43	
Is your child still breastfeeding?	Yes	140(13.6)	110(72.6)	1		0.136
	No	886(86.4)	643(27.4)	0.72	0.47–1.11	
Child received feeding at < 6 months of age?	Yes	415(39.4)	293(70.6)	1		0.133
	No	639(60.6)	478(74.8)	1.24	0.94–1.63	
Has the child taken vitamin A&D drops?	Yes	899(86.1)	655(72.9)	1		0.448
	No	145(13.9)	110(75.9)	1.17	0.78–1.76	
Is the child currently taking the drops?	Yes	108(12.1)	80(74.1)	1		0.745
	No	788(87.9)	572(72.6)	0.93	0.59–1.47	
Where does the child receive the drops?	MOH	437(48.7)	322 (73.7)	1		0.444
	UNWRA	325(36.2)	227(69.9)	0.83	0.60–1.14	
	NGOs	15(1.7)	11(73.3)	0.98	0.31–3.15	
	Private	117(13.0)	90(76.9)	1.19	0.74–1.92	
	Medical Missions	3 (0.3)	3(100)	N/A	N/A	

^a^Unadjusted odds ratios from bivariable statistical analyses

^b^*p*-value from chi-squared test unless otherwise stated

While there was no trend detected in age nor differences between the genders, there was a strong association found between higher levels of maternal education and lower levels of VAD in children. Furthermore, there was a large statistically significant variation in deficiency rates of vitamin A between and within the West Bank and Gaza Strip (see footnote Table 3 for classification of regions). For instance, the highest and lowest mean levels of vitamin A were found in the North West Bank (WB) in Tubas and Tulkram respectively. Additionally, although confidence intervals were very wide, children in the Bedouin camps were over five times more likely to have VAD than children living in cities. Contrastingly, their counterparts in the refugee camps were 26% less likely to have VAD than children living in cities. While not found to be statistically significant, this may correlate to the result that children who received drops from the UNRWA were 17% less likely than children receiving drops from the MOH to have VAD. There was no evidence of an association between breastfeeding and complementary feeding and vitamin A deficiency.

**Table 3:** Unadjusted Associations between Risk Factors and Vitamin D Deficiency

Risk Factor	Subcategory	N (%)	Deficient N (%)	OR*	95% CI	*p*-value^c^
Total	*Total*	150	91(60.7)	N/A	N/A	N/A
Age Group	< 1	23(15.4)	13(56.5)	1		Test-for- trend: 0.836
	1–2	34(22.8)	23(67.7)	1.61	0.54–4.80	
	2–3	35(23.5)	17(48.6)	0.73	0.25–2.10	
	3–4	35(23.5)	24(68.6)	1.68	0.56–4.99	
	4+	22(14.8)	13(59.1)	1.11	0.34–3.63	
Gender	Male	77(51.3)	42(54.6)	1		0.114
	Female	73(48.7)	49(67.1)	1.70	0.88–3.30	
Season of Birth	Winter(Oct-Mar)	76(51.0)	44(57.9)	1		0.523
	Summer(Apr-Sept)	73(49.0)	46(63.1)	1.24	0.64–2.39	
Mother’s Education	Illiterate/Elementary	5(3.4)	3(60.0)	1		Test-for trend: 0.268
	Preparatory/Secondary	85(57.4)	56(65.9)	1.29	0.20–8.14	
	Diploma/BA/Postgrad	58(39.2)	32(55.2)	0.82	0.18–5.28	
Province	West Bank	75(50.0)	42(56.0)	1		0.242
	Gaza Strip	75(50.0)	49(65.3)	1.48	0.77–2.86	
Regions^a^	North WB	32(21.3)	17(53.1)	1		0.206
	Middle WB	17(11.3)	11(64.7)	1.61	0.481–5.44	
	South WB	26(17.3)	14(53.9)	1.03	0.36–8.52	
	North Gaza	35(23.3)	27(77.1)	2.97	1.04–8.52	
	South Gaza	40(26.7)	22(55.0)	1.09	0.42–2.74	
Type of Community	City	55(36.7)	37(67.3)	1		0.213
	Village	46(30.7)	23(50.0)	0.49	0.22–1.09	
	Camp	46(30.7)	30(65.2)	0.91	0.40–2.09	
	Bedouin camp	3(2.0)	1(33.3)	0.24	0.02–2.86	
Has your child ever been breastfed?^b^	Yes	148(98.7)	91(61.5)	N/A	N/A	Fishers exact: 0.153
	No	2(1.3)	0	N/A	N/A	
Is the child still breastfeeding?	Yes	25(16.9)	16(64.0)	1		0.776
	No	123(83.1)	75(61.0)	0.88	0.36–2.15	
Child received feeding at < 6 months of age?	Yes	57(38.0)	32(56.1)	1		0.375
	No	93(62.0)	59(63.4)	1.35	0.69–2.66	
Has the child taken vitamin A&D drops?	Yes	132(89.2)	76(57.6)	1		Fishers exact: 0.003
	No	16(10.8)	15(93.8)	11.1	1.42–86.2	
Is the child currently taking the drops?	Yes	16(12.2)	5(31.3)	1		0.025
	No	115(87.8)	70(60.9)	3.42	1.11–10.5	
Where does the child receive the drops?	MOH	64(48.5)	38(59.4)	1		Fishers exact: 0.927
	UNWRA	37(28.0)	20(54.1)	0.80	0.36–1.82	
	NGOs	1(0.8)	1(100)	1	N/A	
	Private	30(22.7)	17(56.7)	0.89	0.37–2.15	
	Medical Missions	0	0	N/A	N/A	

*Unadjusted odds ratios from bivariable statistical analyses

^a^Districts grouped into regions due to small numbers. North West Bank (WB): Jenin, Tulkram, Nablus, Qalqiliya, Salfit, Tubas; Middle West Bank: Ramallah & Al-Bireh, Jericho & Aghwar, Jerusalem; South West Bank: Bethlehem, Hebron, South Hebron; North Gaza: North Gaza, Gaza; South Gaza: Deir El Balah, Khan Yunis, Rafah

^b^No OR due to collinearity

^c^*p*-value from chi-squared test unless otherwise stated

### Vitamin D results

For the subsample of children that were examined for vitamin D (*n* = 150) the mean level of the micronutrient was 47.6 nmol/L (StDev: 17.0) and 60.7% of children were considered to be deficient (95%CI 52.8–68.4%). The unadjusted odds ratios associated with each level of a risk factor are illustrated in Table 3.

Similar to Vitamin A, there was no statistically significant association between VDD and increasing age, season of birth, practice of breastfeeding and complementary feeding nor gender. Although not found to be statistically significant, similarly to vitamin A, children who received drops from the UNRWA were 20% less likely to have VDD than children receiving the supplements from the MOH. However, unlike vitamin A, there was no evidence of an association of between VDD region of residence, maternal education level nor type of community).

### Relationship between vitamin A and D3 levels and haemoglobin levels

There were 1124 observations of haemoglobin (Hb) levels (Table 4). The mean level of Hb in the sample was 11.6 g/dl (StDev: 1.11). Those children with anaemia were 47% more likely to have VAD (*p* = 0.027). There was no statistical evidence of an association between vitamin D deficiency and anaemia.

**Table 4:** Association between Categorical Levels of Vitamin A and D and Anaemia

Vitamin	Risk Factor	Subcategory	N	Deficient N (%)	OR	95% CI	*p*-value
Vitamin A	Hb Level	*Total*	984	722 (73.4)			0.027
		Hb > 11.0 g/dl	739	529 (71.6)	1		
		Hb < 11.0 g/dl	245	193 (78.8)	1.47	1.04–2.08	
Vitamin D	Hb Level	*Total*	145	91(60.7)			0.550
		Hb ≥ 11.0 g/dl	100	63(63.0)	1		
		Hb < 11.0 g/dl	45	26(57.8)	0.804	0.39–1.65	

### Multiple logistic regression analysis

#### Vitamin A results

After adjusting for the other factors listed in Table 5, most risk factors showed no statistical association with VAD, with the exception of three variables. Similar to the bivariable analyses, those with anaemia were 50% more likely to be deficient in vitamin A. Children who did *not* receive complementary feeding before 6 months of age were 53% more likely to have VAD. Increased levels of CRP remained associated with an increased odds of deficiency. Furthermore, a mother’s education was no longer a significant predictor of deficiency after adjusting for all other factors.

**Table 5:** Results from Multiple Logistic Regression Analysis for Vitamin A

Risk Factor	Subcategory	Unadjusted OR	95% CI	p-value^c^	Adjusted OR^a^	95% CI	*p*-value
Age Group	< 1	1		Test-for trend: 0.299	1		0.305
	1–2	0.99	0.63–1.58		1.13	0.53–2.42	
	2–3	1.10	0.69–1.76		1.65	0.94–2.89	
	3–4	0.94	0.58–1.53		0.99	0.52–1.90	
	4+	1.38	0.82–2.31		1.18	0.61–2.25	
Gender	Male	1		0.916	1		0.689
	Female	1.01	0.77–1.33		0.93	0.68–1.28	
Mother’s Education	Illiterate/Elementary	1		Test-for- trend: 0.008	0.803^b^	0.56–1.15	0.154
	Preparatory/Secondary	0.77	0.40–1.48				
	Diploma/BA/Postgrad	0.54	0.27–1.05				
Province	West Bank	1		0.914	1		0.183
	Gaza Strip	0.99	0.75–1.29		1.34	0.78–2.31	
Type of Community	City	1		0.058	1		0.126
	Village	0.82	0.59–1.13		0.76	0.48–1.20	
	Camp	0.74	0.53–1.04		1.01	0.51–1.99	
	Bedouin camp	5.54	0.73–42.1		4.74	2.21–10.2	
Has the child ever been breastfed?	Yes	1		0.283	1		0.224
	No	0.65	0.30–1.43		0.56	0.25–1.22	
Child received feeding at < 6 months of age?	Yes	1		0.133	1		0.015
	No	1.24	0.94–1.63		1.53	1.18–1.98	
Is the child currently taking the drops?	Yes	1		0.745	1		0.854
	No	0.93	0.59–1.47		0.95	0.58–1.55	
How long has the child taken the drops? (months)	Continuous Variable	1.03	0.99–1.06	0.142	1.03	0.99–1.07	0.167
Where does the child receive the drops?	MOH	1		0.444	1		0.126
	UNWRA	0.83	0.60–1.14		0.59	0.30–1.18	
	NGOs	0.98	0.31–3.15		0.58	0.09–3.61	
	Private	1.19	0.74–1.92		1.00	0.54–1.85	
	Medical Missions	N/A			N/A		
CRP (mg/L)	Continuous Variable	1.02	1.00–1.04	0.001	1.02	1.00–1.03	0.016
Anaemia	Yes	1.47	1.04–2.08	0.027	1.50	1.08–2.10	0.047
	No	1			1		

^a^Adjusted odds ratios adjusting for all other factors in the table. Likelihood Ratio Tests provided p-values

^b^Mother’s education was treated as a continuous variable as the variable was found to be statistically significant in a test for trend in the bivariable analyses

^c^*p*-value from chi-squared test unless otherwise stated

There were five risk factors with adjusted odds ratios that were not statistically significant but the trends may be worth exploring in future research. Those children living in the Gaza Strip, males and older children (with the exception of children aged 3 to 4) were more likely to have VAD. Additionally, the children who receive the drops from the UNRWA and NGOs appear less likely to have VAD as opposed to those children who are supplied by the MOH.

#### Vitamin D results

Adjusted odds ratios in Table 6 highlight four risk factors that demonstrate statistical significance. There is a trend in age, with an exception of children aged 2 to 3, those children who are older are more likely to be deficient in this micronutrient (*p* = 0.0429). The results establish that females have a 2.72 higher odds of deficiency than males. The level of CRP in the serum is associated with the level of vitamin D (*p* = 0.031). Those children who were not taking the drops at the time of the survey have approximately 5 times higher odds of deficiency than those child taking the drops at the time of the survey, although, it is important to note the wide confidence interval (95%CI 1.11–24.5).

**Table 6:** Results from Multiple Logistic Regression Analysis for Vitamin D

Risk Factor	Subcategory	Unadjusted OR	95% CI	*p*-value	Adjusted OR^a^	95% CI	*p*-value^1^
Age Group	< 1	1		Test-for-Trend: 0.836	1		0.043
	1–2	1.61	0.54–4.80		1.11	0.29–4.16	
	2–3	0.73	0.25-2a.10		0.24	0.06–0.97	
	3–4	1.68	0.56–4.99		1.61	0.19–16.8	
	4+	1.11	0.34–3.63		1.28	0.23–7.26	
Gender	Male	1	0.88–3.30	0.114	1		0.037
	Female	1.70			2.72	1.21–6.01	
Season of Birth	Winter (Oct-Mar)	1		0.523	1		0.913
	Summer (Apr-Sept)	1.24	0.64–2.39		1.06	0.30–3.76	
Mother’s Education	Illiterate/Elementary	1		Test-for-Trend: 0.268	N/A		0.347
	Preparatory/Secondary	1.29	0.20–8.14		1		
	Diploma/BA/Postgrad	0.82	0.18–5.28		0.62	0.28–1.36	
Province	West Bank	1		0.242	1		0.203
	Gaza Strip	1.48	0.77–2.86		1.96	0.67–5.71	
Type of Community	City	1		0.213	1		0.715
	Village	0.49	0.22–1.09		0.67	0.20–2.26	
	Camp	0.91	0.40–2.09		1.24	0.25–6.15	
	Bedouin camp	0.24	0.02–2.86		0.27	0.10–0.74	
Child received feeding at < 6 months of age?	Yes	1		0.375	1		0.824
	No	1.35	0.69–2.66		0.89	0.25–3.20	
Is the child currently taking the drops?	Yes	1		0.025	1		0.054
	No	3.42	1.11–10.5		5.23	1.11–24.6	
How long has the child taken the drops? (months)	Continuous Variable	1.00	0.92–1.08	0.989	1.02	0.89–1.17	0.768
Where does the child receive the drops?	MOH	1		Fishers exact: 0.927	1		0.457
	UNWRA	0.80	0.36–1.82		0.70	0.15–3.25	
	NGOs	Omit			Omit		
	Private	0.89	0.37–2.15		0.45	0.15–1.37	
	Medical Missions	N/A			N/A		
CRP (mg/L)	Continuous Variable	0.97	0.94–1.00	0.033	0.96	0.93–0.99	0.031
Anaemia	Yes	0.804	0.392-1.65	0.550	1.16	0.44–3.08	0.780
	No	1			1		

*Adjusted odds ratios by all factors in the table. Likelihood Ratio Tests provided p-values

^1^*p*–value from chi-squared test unless otherwise stated

While not statistically significant, the following findings from the adjusted analysis may help inform future research. Those children living in camps or in the Gaza Strip or who were anaemic were more likely to be deficient in vitamin D. As for health service provider, the odd ratios depreciated indicating a stronger adjusted relationship with receiving drops from the UNRWA or private clinics and lower levels of deficiency as compared to children receiving drops from the MOH.

## Discussion

### Understanding the burden of disease

The evidence suggests that the State of Palestine has a higher prevalence of vitamin A and D deficiencies than some of its neighboring countries. Vitamin A deficiency was found in 73.1% of children, which is similar to the 76% prevalence rate found in two previous cross-sectional studies in the State [14, 24], however, it is much larger than the 15% to 40% range observed in Southern Israel, Turkey and Iran [16, 20, 21, 25]. As for vitamin D, 60.7% of children were deficient, which falls in the upper end of the range of deficiency reported by Bassil et al. in a systematic review of VDD in the Middle East and North Africa (30–75%) [3]. As for comparison on a global scale, prevalence of VDD among children in the United States and the UK is 16% and 35% respectively [26]. In most western countries, VAD is not considered a public health concern, however, prevalence estimates of 44%, 45% and 21% in South Asia, Sub-Saharan Africa, and Latin American and the Caribbean respectively serve as poignant comparison to the 33% deficiency cut-off (< 0.70 μmol/L) observed in this study [27]. Thus, Palestine is a unique state in the region, and supplementation programs must be designed for its own population. There are three potential fronts for improvements: (1) target populations that are at higher risk of these micronutrient deficiencies; (2) encourage continuous uptake of vitamin A and D drops through maternal education programs; and (3) ensure the adequate supply of supplements to health service providers.

### Targeting at-risk populations

#### Older ages

As found in a study by USAID, while a trend is observed, there was no statistical association that older aged children have higher odds of VAD than children under one [14]. However, a Jordanian study supports the strong evidence of an association found with increasing age and VDD [5]. Children receiving drops from the MOH are only being provided supplements in the first 12 months of life, and are subsequently dependent on their parents to provide them with micronutrient rich diets. However, especially in Gaza, access to and parental knowledge of these foods may be limited. It is essential that information be provided on the importance of provision of vitamin A and D rich foods after the supplementation period.

#### Gender differences

USAID’s study in Palestine similarly did not find a strong statistical association of VAD and gender [14]. However, strong evidence of gender differences was found in a study in Southern Israel, where males were almost 4 times more likely to be deficient (OR: 4.17, 95%CI 1.14–15.32 *p* = 0.031) [16]. For VDD the inverse association was found in several studies [11, 17], including a large cross-sectional survey in Jordan, as females were 74% more likely to have VDD than males (95%CI 1.22–2.47 *p* = 0.002) [4]. While further research is warranted, based on the patterns in gender observed, improvements to the supplementation program may entail targeted messages to mothers regarding these unique gender differences.

#### Season of birth

In addition to patterns in age and gender, those children less than one-year-old born in the winter had a lower mean level of vitamin D compared to children in the same age group born in the summer. While there was no evidence of an association in the present study, work from Iran and Jordan found neonates born in the winter to have a 5.7 times (95% CI 2.1–15.7 *p* < 0.01) and a 2.34 times (95%CI 3.13–1.49 *p* < 0.001) higher odds of VDD than neonates born in the summer [6, 8]. Further research may examine if promotion campaigns for vitamin D may be beneficial during winter months.

#### Practices of breastfeeding and complementary feeding

Our results suggest that breastfeeding is not a significant predictor for deficiency. This was a common finding from other studies for both vitamin A [16, 21] and vitamin D [4, 5]. It is understandable that it is not a significant risk factor for VDD as the primary sources of this vitamin are UV radiation and food intake. However, for vitamin A it has been suggested that breastmilk does provide an adequate amount of this micronutrient for children [14]. An association may not have been found in this study because the majority of children in the survey were no longer breastfeeding.

The second dietary risk factor examined was the practice of complementary feeding. It is not recommended until after six months of exclusive breastfeeding to prevent infections. Thus, children who are complementary fed before the age of six months may be more prone to infections and subsequently suffer depleted serum micronutrient levels. Although the results were not statistically significant, the adjusted analysis revealed that the children who were *not* complementary fed before six months were *less* likely to be deficient in vitamin D. However, this does not appear to be the case for vitamin A. Those children who were *not* complementary fed before 6 months were *more* likely to be deficient in vitamin A. It could be hypothesized that those children who were provided food before six months, were given vitamin A rich food. There is limited research on complementary feeding’s role in deficiency in this region, however, collecting more data may be crucial to better understanding the high prevalence of deficiency in the region.

#### High CRP levels and anaemic children

Simultaneous bodily infections and ailments must be taken into consideration when defining at-risk populations for deficiency. Increased levels of CRP were highly correlated to increasing levels of vitamin A and D deficiency in the adjusted analyses. Thus, those children who are more prone to infections like diarrhoea are more likely to have micronutrient deficiencies. Similarly, children with anaemia were found to be at a higher risk of VAD deficiency which is evidenced in other studies [13, 14]. While, children with anaemia were 16% more likely to have VDD, the adjusted analysis demonstrated no evidence of an association which is similar to the finding by Nichols et al., where anaemic children were 30% more likely to be deficient than those children who have normal haemoglobin levels (95%CI 0.94–1.82 *p* = 0.117) [4]. Thus, based on the high correlation to levels of CRP and statistical association between VAD and anaemia, it is essential that children are adequately supplied with supplements and micronutrient rich foods especially if they have pre-existing infections or diseases.

### Encouraging uptake of supplements

It is crucial that each of these high risk groups are targeted to ensure adequate uptake of supplements. To achieve this, a child’s mother must be involved, as she is one of the most important figures to help a child achieve sufficient levels of these micronutrients. She is responsible for providing a child with the supplements for the first 12 months of life. The data suggests that the majority of children are provided these supplements, however, they are not provided for the full 12-month regimen recommended by the MOH. In Gaza the average duration of the regimen was 4.43 months and in the West Bank it was 8.87 months.

Despite no evidence of statistical significance in the adjusted analysis for both VDD and VAD, similar to this analysis, a Jordanian study found children of mothers with lower levels of education were 21% more likely to have VDD (95%CI 0.87–1.69) [4]. Coles et al. determined that a mother with higher education was less likely to have a child with a VAD (OR 0.81 95%CI 0.68–0.95 *p* = 0.011) [16]. Neither of these studies explored the specific practices more educated mothers employed to ensure their children maintained healthy levels of vitamins A and D. Further exploration of these practices may inform the development of maternal education programs which may be a solution to increasing uptake of vitamin supplements.

### Improving health service provision

The last step required to improving rates of deficiency is targeting health service provision networks. While not found to be statistically significant, children who received the vitamin A and D drops from any other organization than the MOH were less likely to be deficient. There are two potential explanations for these differences: supplement availability (Table 7) and differences in the regimen recommendations by health service providers. As seen in Table 7, those who received drops from the UNRWA stated that the supplement was always available 92.6% of the time (compared to only 85.2% of the time at the MOH), and its children (primarily refugees living in camps) have lower levels of deficiency. At first, this result may be perplexing since the UNRWA supplies 60% of services in Gaza which has higher odds of vitamin A and D deficiency than the West Bank. First, it is important to note that at the time of the study, UNICEF supplied vitamin A and D drops to both the MOH and UNRWA. Therefore, since the supplier (UNICEF) was the same for both organizations, the differences in deficiency levels among children may be better attributed to the variations in protocols between the two service providers, MOH and UNRWA. The UNRWA provides vitamin A capsules to children up to the age of five years, whereas the MOH only supplies the supplement for the first year of a child’s life. Furthermore, the UNRWA provides systematic counseling services to mothers of children less than five years for better uptake of vitamin A and D. Thus, the more comprehensive supplementation program by the UNRWA may warrant further investigation to decrease levels of VAD and VDD in the State of Palestine.

**Table 7:** Availability of Vitamin A & D Drops by Health Service

Health Service	Available Always N (%)	Available Sometimes N (%)	Not Available N (%)
MOH	465 (85.2)	79 (14.5)	2 (0.37)
UNRWA	376 (92.6)	28 (6.90)	2 (0.49)
NGOs	16 (84.2)	3 (15.8)	N/A
Private	122 (84.9)	20 (14.1)	N/A
Medical Missions	N/A	4 (100)	N/A

### Strengths and limitations

The nature of the cross-sectional study design inhibits any inferences of causal association. Compounded with the small sample of vitamin D, there may have been selection bias that would reduce the generalizability of the results to the entire population. Children were recruited from health clinics, which some children, especially in areas of Gaza, are unable to access for various reasons. While the analysis adjusted for CRP, no other co-morbidities were assessed, and those children presenting at the clinic may also have been more likely to have presented with existing infections, and thus have been more likely to be deficient in micronutrients.

The pseudo-R-squared values from the multiple regression models were relatively low, 0.0413 and 0.1900 for vitamin A and D respectively. This may be explained by the fact that we were unable to assess additional confounders such as measures of other forms of malnutrition like stunting and wasting. Detailed data on the average daily intake of certain foods was not added into the model. Since a crucial source of vitamin A and D is intake of micronutrient rich foods, measuring this factor could help explain the high levels of deficiency.

Lastly, the majority of the results were statistically insignificant at the 5 % level as many confidence intervals around estimates included one. As the prevalence value used to calculate the sample size was for anaemia and not vitamin A and D deficiencies the results may have been underpowered. While results did not often reach levels of statistical significance, the authors justified their inclusion to help inform future research.

## Conclusions

The prevalence of vitamin A and D deficiency in Palestine is relatively high compared to some of its neighboring countries. Due to the complex economic and political situation in the country, it is critical to examine risk factors for these micronutrient deficiencies. While the uptake of supplementation is high, few children receive the full regimen, and there are huge variations in deficiency levels in the different regions of the State. Health service provision pathways may be key in ensuring uptake of the supplements and lowered deficiency levels. A deeper understanding of the UNRWA efforts, including the counselling services to mothers and the prolonged supplementation period (for vitamin A), is needed. If this program is proven effective, it may serve as a model for the entire State. The counselling for mothers may include advice on maintaining micronutrient levels of high risk groups of children particularly those with comorbidities such as anaemia and may inform them of the micronutrient rich foods she must supply her child after the supplementation regimen.

While the study examines the most common risk factors for vitamins A and D, there remains a need for further large representative surveys especially for risk factors for vitamin D due to the smaller sample size. Subsequent studies should include an in-depth review of daily nutrient intake to permit better understanding of food behaviour and its influence on deficiency.

## Abbreviations

CI: Confidence Interval
CRP: C - reactive protein
GZ: Gaza Strip
Hb: Haemoglobin
MOH: Ministry of Health
NGOs: Non-Governmental Organizations
OR: Odds Ratio
PMS: Palestinian Micronutrient Survey
StDev: Standard Deviation
UNRWA: United Nations Relief and Works Agency for Palestine Refugees
VAD: Vitamin A Deficiency
VDD: Vitamin D Deficiency
WB: West Bank
WHO: World Health Organization
